# Electrochemical Impedance Spectroscopy Analysis of Organic Epoxy Coatings Reinforced with Nano Clay

**DOI:** 10.3390/ma17123028

**Published:** 2024-06-20

**Authors:** Davide Asperti, Marina Cabrini, Sergio Lorenzi, Giuseppe Rosace, Abdollah Omrani, Tommaso Pastore

**Affiliations:** 1 Department of Engineering and Applied Sciences, School of Engineering, University of Bergamo, 24044 Dalmine, Italy; sergio.lorenzi@unibg.it (S.L.); giuseppe.rosace@unibg.it (G.R.); tommaso.pastore@unibg.it (T.P.); 2 Faculty of Chemistry Iran, University of Mazandaran, Babolsar 4741613534, Iran; omrani@umz.ac.ir

**Keywords:** corrosion, protection, organic coating, nanoparticles, barrier effect, electrochemical impedance spectroscopy, equivalent electrical circuit, cluster

## Abstract

Electrochemical impedance spectroscopy (EIS) is a modern and efficient method for the evaluation of the protective abilities of coatings. However, the interpretation of the experimental data is a difficult task. This paper aims to investigate the effect of the addition of a nano clay, Cloesite 30B^®^, on the barrier properties of an epoxy-based system through electrochemical impedance spectroscopy in an aerated sodium chloride solution. The EIS spectra of the samples analysed showed different evolutions over time. The subsequent processing of spectra using equivalent electrical circuits is an excellent analytical tool and allows the protective capacity of coatings to be assessed. By using this analysis, it was possible to define and comprehend the impact of adding nano clay in different concentrations to the epoxy resin coating. The work has shown the effectiveness of increasing the barrier effect of the coating with this type of nano clay. However, the improvement is linked to obtaining a correct dispersion of nanoparticles. Otherwise, there is the formation of macro-clusters of particles inside the coating. Their appearance can cause a deterioration in coating performance.

## 1. Introduction

A growing focus is guiding the research into the use of increasingly sustainable and effective solutions for corrosion protection methods. Increasing the sustainability of organic coatings involves the extension of their maintenance over time and the use of fillers with less environmental impact [1,2].

In recent years, innovation in the field of organic coatings has mainly focused on improving the barrier effect through fine and ultra-fine pigments and fillers. Mathiazhagan [3] reports on enhancing properties by additions of fillers with dimensions ranging from micrometric to nanometric. Well-dispersed nanoparticles within the polymer matrix can enhance the contribution of the filler–binder interface to oxygen diffusion and water uptake [4]. Nanofillers of different natures—both organic and inorganic—have been taken into account to allow the realisation of coatings with better barrier properties, covering Si [5], SiO_2_ [6], Al [7], Al_2_O_3_ [8], TiO_2_ [9], Zn [7], ZnO [10], ZrO_2_ [11], Fe_2_O_3_ [12], Carbon nanotubes [13], Graphene, and Graphene Oxide [14]. Clay nanoparticles [15,16,17,18] are an interesting alternative solution due to their low cost and easy availability [15,19].

Previous studies demonstrated that nano clays can improve corrosion protection, also enhancing the mechanical properties of coatings. Olivier et al. report that improving coating performance depends on size distribution, exfoliation state, dispersion, and concentration of particles [20].

The most widely used clays belong to the 2:1 phyllosilicate family, which have a crystalline lattice with a central layer of alumina or magnesia enclosed between two layers of silica [21]. This family includes montmorillonite, a type of clay widely used as a nanofiller for organic coatings [22]. This type of clay has good cation exchange capacity (CEC), which gives it a high dispersion capacity within the polymer matrix [23]. Its use in nanometric form can further increase the surface area of interaction with the polymer matrix, thus increasing the barrier of the coating even with low-quantity additions of nanoparticles, less than 5% by weight [24].

Azeez et al. [25] outline that, in order to achieve good barrier properties, mechanical and thermal properties are essential for a high degree of interaction between nanofillers and the polymer matrix [25], which may be obtained by treatments able to modify the hydrophilic nature of clay minerals to make it organophilic, according to Sinha Ray and Okamoto [26]. Such treatments can enhance the compatibility of nanoparticles with the polymer matrix and their dispersion [26].

Electrochemical impedance spectroscopy (EIS) enables the assessment of the coating’s behaviour during the exposure period. In particular, the EIS spectra evolution provides the opportunity to make hypotheses about the mechanism of coating degradation. Le Thu et al. [27] used the evolution of EIS spectra and an electrical equivalent circuit to analyse the coating capacity and the water absorption inside the coating. Harvey et al. [28] used the impedance modulus evolution to estimate the electrochemically active area on epoxy coatings.

The present work employs the use of a montmorillonite nano clay, Cloisite 30B^®^. It was chosen because it could be used inside a coating and had acceptable costs for industrial painting. The paper reports on exposure tests of steel specimens protected by epoxy-based coatings to assess the effect of organo-modified montmorillonite nanoparticles. Electrochemical impedance spectroscopy monitored the corrosion behaviour of coated specimens for up to 1000 h of immersion in aerated chloride solutions.

## 2. Materials and Methods

**Figure 1:**
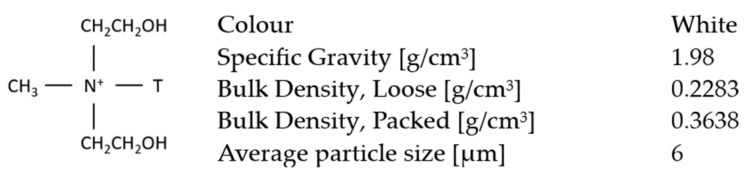
Chemical structure and physical properties of Cloisite 30B^®^.

The tests were performed on Cloisite 30B^®^ nano clay provided by Southern Clay Products Inc. (Louisville, KY, USA). Cloisite 30B^®^ was chosen because it could be used inside a coating and had acceptable costs for industrial painting. The nano clay is a quaternary-ammonium-salt-modified natural montmorillonite nano clay, shown in Figure 1. The morphology and dimensions of particles were evaluated by scanning electron microscope (SEM) (Zeiss Gemini, Jena, Germany) and Malvern Mastersizer 3000E (Malvern, UK) granulometer equipped with an Aero unit.

The coating was performed using DLVE18^®^ commercial epoxy resin and DEH4911^®^ hardener, manufactured by Olin Corporation (Clayton, MO, USA), with a resin/hardener ratio of 100/49. The nanoparticles were added to the coating at concentrations of 1%, 3%, and 5% by weight.

The dispersion procedure involved two phases. In the first step, the nano clay was added to the resin and maintained in an ultrasonic bath for 1 h. Following the addition of the hardener, mixing by mechanical stirrer was maintained for 5 min, and by ultrasonic bath for a further 30 min.

The specimens consisted of 60 × 90 × 1.5 mm carbon steel plates with the following chemical weight composition: 0.051%C, 0.01%Si, 0.332%Mn, 0.01%P, 0.014%S, 0.033%Al.

To ensure uniform surface roughness, the steel surface was treated with sandblasting and pickled in concentrated hydrochloric acid for 300 s, and the surface roughness was measured by a confocal interferometric profilometer.

The coating was then applied using a hand lay-up technique. An amount of resin was deposited at one end of the specimen and then spread over the entire surface of the specimen using a shaped spreader bar to achieve the required thickness. Crosslinking was in air at room temperature.

Both wet and dry film thicknesses were measured. The dry thickness was obtained through magnetic gauge. Table 1 shows the designation of the specimens.

Electrochemical impedance spectroscopy tests were performed according to EN ISO 16773-2:2016, in 3.5% NaCl solution using PMMA electrochemical test cells equipped with a saturated calomel reference electrode (SCE) and an activated titanium counter electrode. Exposed specimen area was 12.57 cm^2^. EIS spectra were obtained from 0.01 Hz to 400 kHz, with 10 measurements per decade and 10 mV voltage amplitude. EIS spectra were measured after 2 h, 70 h, 135 h, 235 h, 330 h, 615 h, and 1000 h of exposure on two specimens for each testing condition.

## 3. Results and Discussion

### 3.1. Morphological Analysis of the Particles

**Figure 2:**
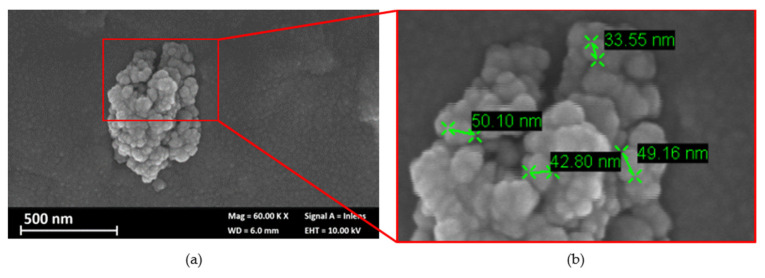
(**a**) SEM image of a macro-cluster of nano clay particles; (**b**) detail of the macro-cluster.

The morphology and dimensions of particles were evaluated by scanning electron microscope and laser granulometer. Figure 2 shows an SEM image of a macro-cluster, which denotes the strong tendency to aggregate of nanoparticles with sizes between 20 nm and 60 nm. The macro-cluster distribution, evaluated with a laser granulometer, was D (10) 3 µm, D (50) 14 µm, and D (90) 35 µm. These results confirm the strong tendency of the nanoparticles to form macro-clusters. Only 10% of the particles analysed were under the dimension of 3 µm, 50% were under 14 µm, and 90% of the cluster was smaller than 35 µm.

### 3.2. Surface Roughness and Coating Thickness

**Figure 3:**
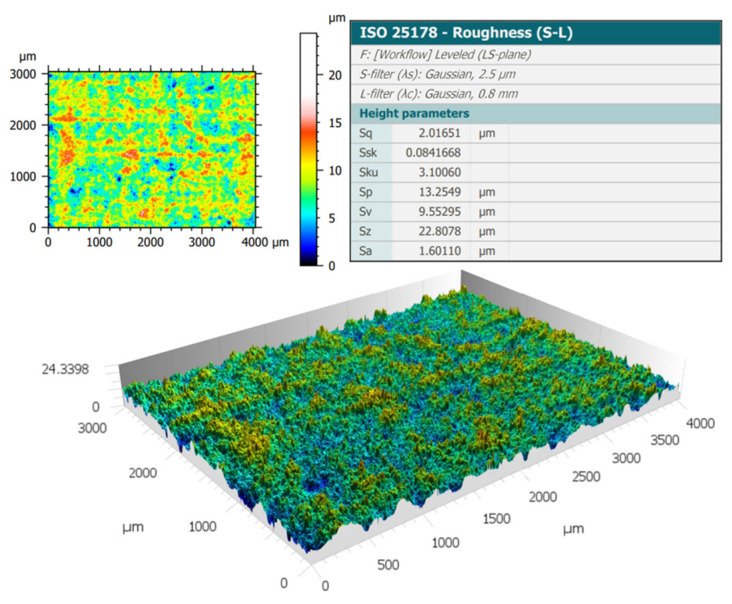
Roughness analysis of pickled surface by confocal interferometric profilometer.

Before coating application, the roughness of the substrate was assessed after surface preparation of specimens by pickling, obtaining values of maximum peak height (Sz) according to ISO 25178 [29] less than 23 µm in all cases (Figure 3). The surface roughness value is not influenced by the sample. All samples received the same surface preparation. The surface preparation was chosen to permit the application of a thin layer of coating, to accelerate the corrosion tests but always keep the peaks covered by the coating.

Table 1 summarises the wet and dry thickness measurements. A nominal wet thickness of 75 µm was applied, producing a dry layer of 52 µm to 56 µm. Only for the C1 and D2 specimens was the dry thickness 39 and 44 µm, respectively, but it was still greater than the maximum height of the roughness peak.

### 3.3. Morphological Analysis of the Corrosion Attacks

**Figure 4:**
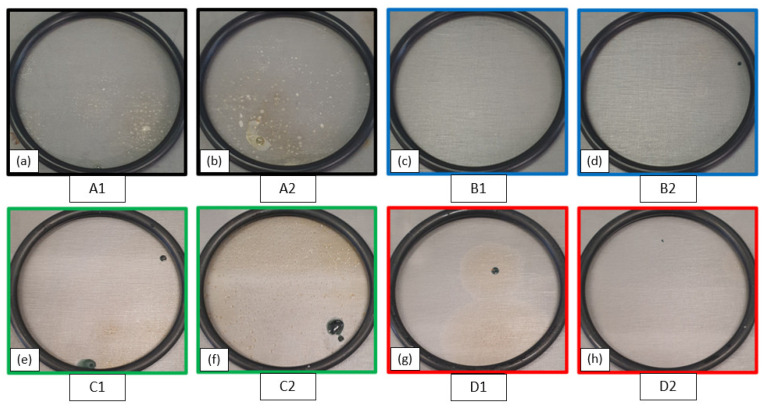
Surfaces of specimens after 1000 h of immersion in NaCl 3.5% solution; (**a**,**b**) Specimens with epoxy resin coating; (**c**,**d**) Specimens with 1 wt% of nanoclay; (**e**,**f**) Specimens with 3 wt% of nanoclay; (**g**,**h**) Specimens with 5 wt% of nanoclay.

After exposure to 3.5% NaCl solution for 1000 h, the surfaces of the specimens were observed in order to assess alterations. Figure 4 shows the modification of the aspect that occurred on the exposed surface after 1000 h.

The analysis of the specimens’ surfaces allows us to divide the behaviours into different types, without any correlations with the initial content of nano clay. In the first case, blistering zones are observed, together with a slight change in the colour of the surface without evident corrosion products, shown in Figure 4a,b. In the second case, the surface shows no corrosion products and blistering zones of the coating, shown in Figure 4c. The third case includes those specimens whose surface has evident corrosion products, shown in Figure 4d,h. The last case includes those specimens on whose surface blistering zones of the coating and areas with evident corrosion products can be identified (Figure 4e–g).

These different morphologies are associated with various behaviours in the EIS spectra.

**Figure 5:**
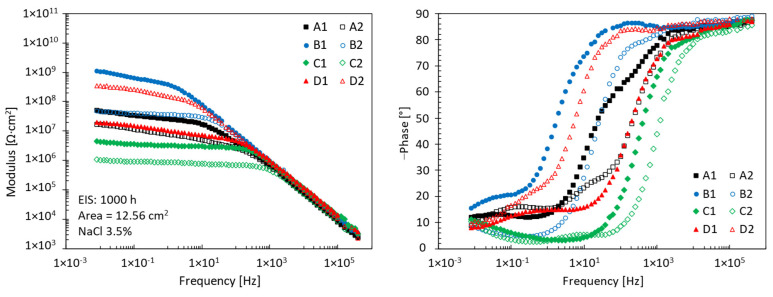
Bode diagrams after 1000 h exposure in 3.5% NaCl solution.

Figure 5 shows all the EIS spectra obtained at the end of the experiment. In the low-frequency area of less than 10 Hz, there is a marked variation of the modulus of impedance between the different samples analysed. The test specimen with the lowest value of modulus is associated with the worst condition. This condition is related to a corrosive attack and an extensive swelling of the coating, which spread in many areas of the surface. The higher impedance modulus is associated with the best condition of those observed. The surface shows no obvious sign of corrosion or swelling of the coating.

### 3.4. Interpolating Equivalent Circuits

The EIS technique allows the resistances and the capacitances in the electrochemical cell to be quantitatively measured. The resistance is related to electron transfer reactions, such as corrosion. The capacitance, for organic coatings, is related to the swelling or to the water uptake, for example. This technique can also monitor the corrosion rate of the metallic substrate, which generally occurs when the protective coatings fail. To quantify these processes, we need to interpolate the EIS spectra with an appropriate equivalent electrical circuit (EEC). This analysis has been extended to all EIS spectra to understand better the evolution of these physical and chemical processes over time.

**Figure 6:**
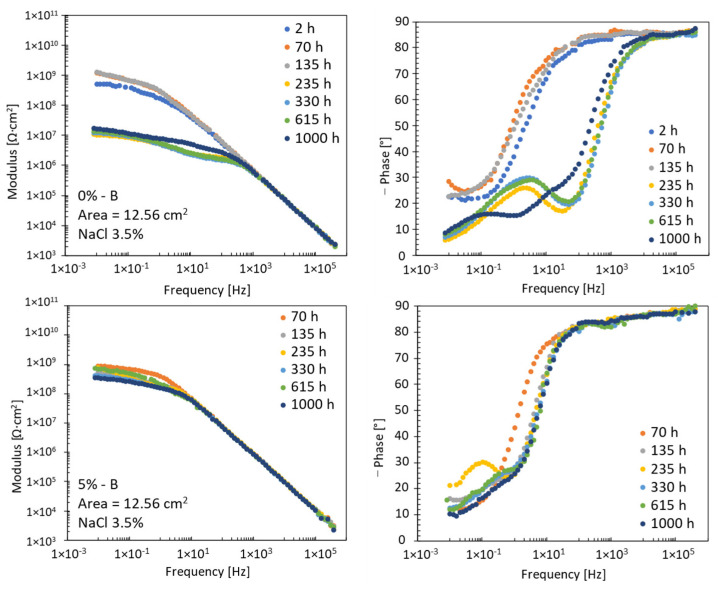
Evolution of EIS spectra during 1000 h exposure in 3.5% NaCl solution.

By extending the time-space of analysis, there is a difference in the evolution of the EIS spectra measured during the 1000 h of exposure. Figure 6 shows, as an example, the evolution of the EIS spectra of two specimens analysed. For some specimens, the tests performed after 2 h of immersion were eliminated because they were unusable for subsequent reworking due to measurement problems.

In the low-frequency zone, the difference in behaviour is very pronounced, while, at high frequencies, there is no substantial difference. As reported by other authors [27,30], the mean value of the impedance modulus in the low-frequency zone can be used to gain a preliminary indication of coating degradation. As from previous observations, different evolutions of the EIS spectra in the low-frequency zone correspond to different morphologies of coating degradation. On the contrary, the high-frequency zone represents the behaviour of the epoxy coating, giving information about swelling or water uptake during the immersion period [31].

**Figure 7:**
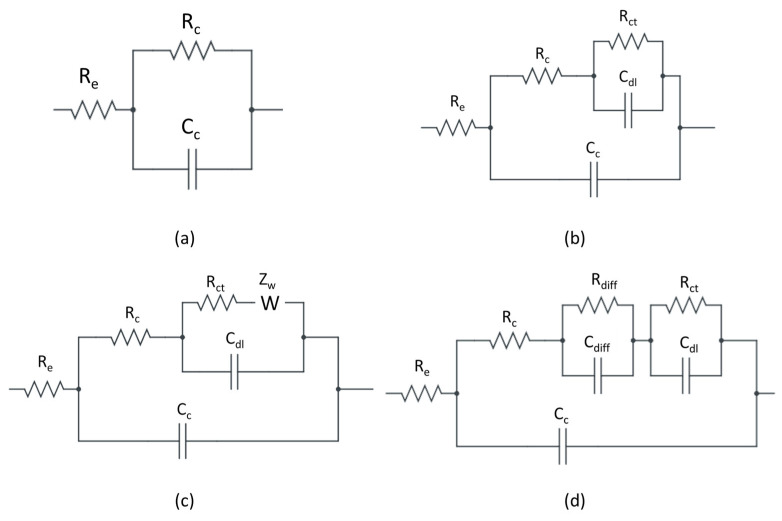
Equivalent electrical circuits used: (**a**) E.C. with one-time constant; (**b**) E.C. with two-time constants; (**c**) E.C. with two-time constants and Warburg impedance; (**d**) E.C. with three-time constants.

To better understand the effect of nanoparticles on the protective capacity of these organic coatings, the EIS tests were analysed using equivalent electrical circuits. As these spectra undergo different evolutions over time, the analysis requires different EECs for each situation. The equivalent circuits used were chosen by analysing the EIS spectra of the various specimens and comparing them with equivalent circuits used in the literature. Figure 7 shows the EECs used [27,32,33]. The first EEC, shown in Figure 7a, was used for specimens that showed intact coating, without water penetration and corrosion phenomena at the interface. R_e_ is the resistance of the electrolyte between the working electrode and the reference electrode. The C_c_ is the capacitance of the coating, while R_c_ is the resistance of the coating [27,34]. In the second EEC, shown in Figure 7b, the R_ct_ is the charge transfer resistance, and C_dl_ is the double-layer capacitance. The addition of these elements in the EEC is connected to the penetration of water inside the coating if the impedance modulus and phase vary in the middle-frequencies range [35]. A variation of impedance modulus and phase in the low-frequency region suggests, instead, that these two elements are connected to the corrosion of the metal substrate [33,36]. In the third EEC, shown Figure 7c, the Z_w_ is the Warburg impedance. This parameter is related to the corrosion reaction controlled by the diffusion process or the mix of the charge transfer and the diffusion process [33]. The resistor R_diff_ and capacitor C_diff_, shown in Figure 7d, are related to the formation of corrosion products on the metal substrate [37]. In the equivalent circuits, a capacitive element is usually shown, but, during the fitting, it is replaced by a constant-phase element, CPE, to compensate for the divergence from the ideal, purely capacitive behaviour, which may be due to the non-homogeneity of the coating thickness [38].

Figure 7 shows that there are elements common to all circuits. These elements are the electrolyte resistance, R_e_, the coating resistance, R_c_, and the coating capacitance, C_c_. Information on the capacitance of the coating during the fitting can be obtained from the high-frequency zone [31]. EIS spectra in the high-frequency region undergo small variations throughout the exposure period. To better understand the evolution of C_c_, the analysis is focused on the range between 1 × 10^4^ Hz and 4 × 10^5^ Hz. This variation over time provides information on the volume of water absorbed within the coating through the Brasher–Kingsbury equation:

ϕ_t_ = log (C_c_/C_c___0_)/log ε_w_ (1)

where C_c_ is the capacity at time t, C_c_0_ is the initial capacity, and ε_w_ is the dielectric constant of water. When water penetrates the interior of the coating, it can no longer be regarded as free water. Its dielectric constant tends to decrease due to the interaction with polar groups in the polymer [38]. Many authors suggest that it varies from a value of 80 to around 50–60 [39]. In this work, the change in coating capacity is used as an indicator of water absorption. If it tends to increase over time, it means that the coating is absorbing water internally.

To perform this analysis, the frequency range was narrowed down to 1 × 10^4^ Hz–4 × 10^5^ Hz. The EIS spectra in this region are thought to display the coating behaviours [40]. For this reason, a single circuit was used for fitting all the experimental curves (Figure 7a).

**Figure 8:**
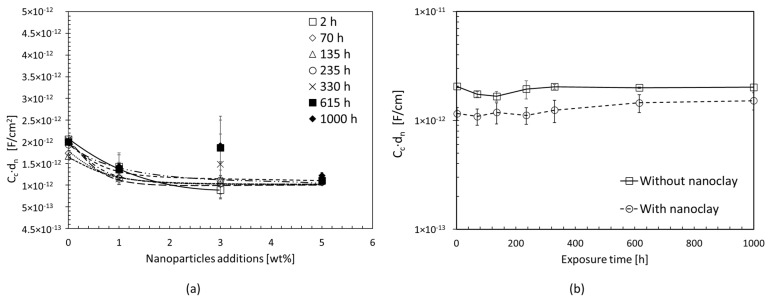
(**a**) Coating capacity, C_c_∙d_n_, during the entire exposure period; (**b**) average coating capacity, C_c_∙d_n,_ of specimens with and without nano clay inside the coating during the entire exposure period.

Figure 8 shows the coating capacity values obtained from processing the EIS spectra. The coating capacity value was calculated by scaling it over the exposed area during the test and multiplying it by the nominal thickness of each coating.

By analysing this parameter throughout the exposure period, it can be concluded that the coating capacity is constant for all the cases studied. Nanoparticles can be added to the epoxy resin to slightly decrease electrical conductivity compared to the epoxy coating (Figure 8a), but it does not affect its performance over time (Figure 8b). During the exposure period, the electrical conductivity of the coating remains constant, resulting in no significant water absorption inside the coating. The coating capacity values stay within the dispersion ranges of the experimental data.

To investigate the influence of nanoparticles on the barrier effect better, the field of analysis is extended to the entire range of frequencies sampled during electrochemical impedance spectroscopy. As noted above, the evolution of EIS spectra changes considerably between the various specimens analysed. It is necessary to use different equivalent circuits for processing the spectra as a function of the observed corrosion morphologies.

**Figure 9:**
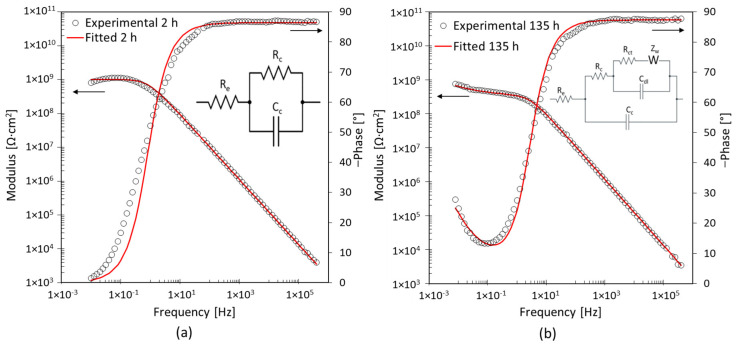
EIS spectra and EEC after (**a**) 2 h and (**b**) 135 h exposure for specimen B1 with 1 wt% of nanoparticles.

In the case of surfaces without corrosion products and blistering zones, shown in Figure 4c, the EIS spectra change significantly after approximately 135 h of exposure. Figure 9 shows the results of the fitting performed on the EIS spectra obtained after 2 h and 135 h of immersion for specimen B1. The Nyquist plots are available in the Supplementary Materials. During the initial instant of immersion, the coating can protect the substrate. The first EEC, shown in Figure 7a, was used to obtain information related to the coating R_c_ and C_c_. During the first 70 h of immersion, the coating’s resistance is enough to assume it can protect the base metal, shown in Table 2. The Rc decreases by an order of magnitude after 135 h of immersion. This reduction is linked to the water penetration through percolation paths inside the coating [34,35]. Simultaneously, the charge transfer resistance, R_ct_, the double-layer capacitance, C_dl_, and the Warburg impedance, Z_w_, are added in the second EEC, as shown in Figure 9b. These elements, in the low frequencies, are associated with corrosion reactions at the interface between the coating and metal substrate [27,41]. The presence of both R_ct_ and C_dl_ as well as Warburg impedance indicates that the corrosion process is driven by both the charge transport mechanism and the diffusion mechanism [33,41].

**Figure 10:**
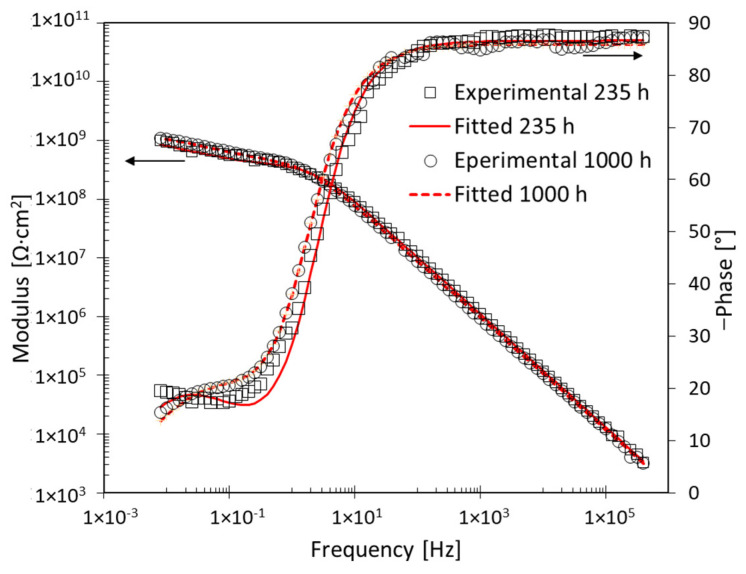
EIS spectra after 235 h and 1000 h exposure for specimen B1 with 1 wt% of nanoparticles.

After 235 h of immersion, shown in Figure 10, the EIS spectra change. It is no longer possible to detect the Warburg impedance. The EEC used for processing this, and the subsequent EIS spectra, consists of only two-time constants, as shown in Figure 7b. After 235 h of immersion, the charge transfer resistance, R_ct_, and the double-layer capacitance, C_dl_, are nearly constant, as shown in Table 2. It can be assumed that the constant trend of R_ct_, the index of corrosion rate, is linked to the presence of corrosion products on the substrate that hinders the corrosion phenomena.

**Figure 11:**
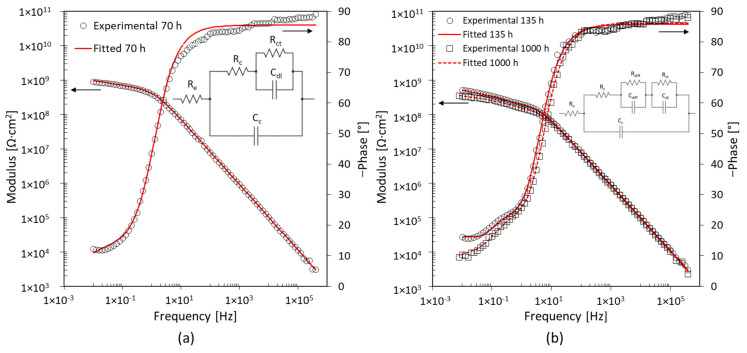
EIS spectra after (**a**) 70 h and (**b**) 135 h exposure for specimen D2 with 5 wt% of nanoparticles.

Figure 11 shows the Bode diagrams after 70 h, 135 h, and 1000 h of immersion for specimen D2. The coating is unable to protect the base metal. The EIS spectrum identifies two-time constants from the initial moments of immersion, shown in Figure 11a. The variation of the impedance modulus occurs in the low-frequency range. Thus, the R_ct_ and C_dl_ are related to the corrosion phenomena at the interface with the base metal [33]. After 135 h of immersion, the third-time constant appears, as shown in Figure 11b. The variation of impedance and phase modulus in the middle-frequency range suggests the creation of a preferential path for water penetration through the coating [34]. The corrosion process of the base metal is still represented by the low-frequency time constant (R_ct_ and C_dl_), Table 3. The third-time constant is related to the middle-frequency range, and it is represented by R_diff_ and C_diff_. The presence of these elements is probably due to the deposit of corrosion products on the base metal [37]. The surface of specimen D2 shows that corrosion products are present, which confirms this assumption, as shown in Figure 4h.

**Figure 12:**
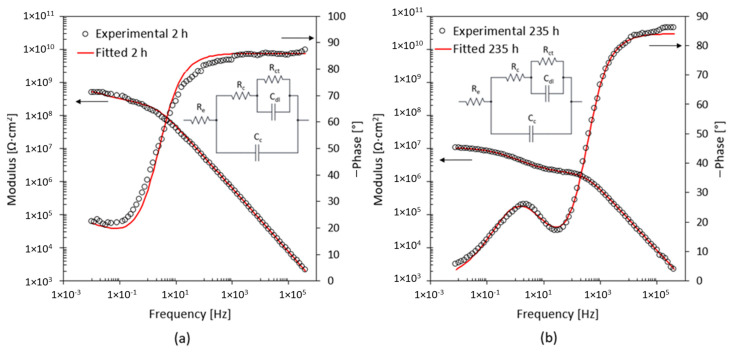
EIS spectra and EEC after (**a**) 2 h and (**b**) 235 h exposure for specimen A2 with 0 wt% of the nanoparticles.

In the case of specimens showing blistering areas of the coating and slight changes in the colour of the surface, shown in Figure 4a,b, the EIS spectrum shows that the coating cannot protect the substrate from the first moments of exposure. By way of example, the most representative ones are shown below. Figure 12 shows the fitting results for the A2 specimen. The coating without nano clays does not protect the metal substrate. In the EIS spectrum after 2 h, the two-time constants, characteristic of a corrosive process already started at the coating–substrate interface, are identified [31]. The equivalent electrical circuit confirms this, due to the presence of the R_ct_ and C_dl_.

**Figure 13:**
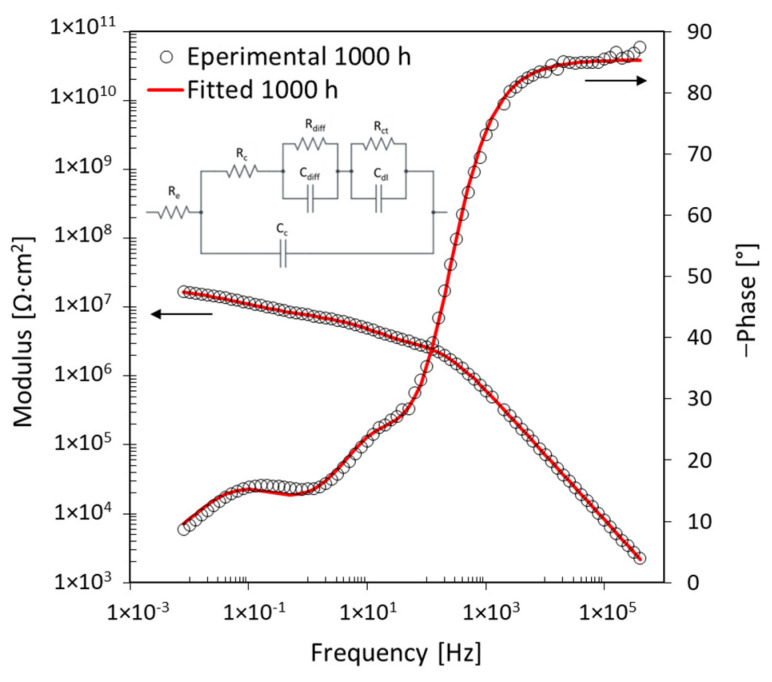
EIS spectra and EEC after 1000 h exposure for specimen A2 with 0 wt% of the nanoparticles.

After 235 h of exposure, there is a variation in the impedance in the middle-frequency zone. The pore resistance, R_c_, decreases by two orders of magnitude, as shown in Table 4. This reduction is related to the formation of the preferential path for the penetration of water through the coating. The coating undergoes a detachment from the substrate due to this phenomenon, as shown in Figure 4a,b. Increasing the exposure time further, up to 1000 h, we see the appearance of the third-time constant, as shown in Figure 13. In the EEC, the resistor R_diff_ and capacitor C_diff_ are added. These elements are associated with the deposit of corrosion products on the substrate [37]. The appearance of this third-time constant makes it possible to explain the origin of the colour change observed on the specimen surface at the end of the test.

**Figure 14:**
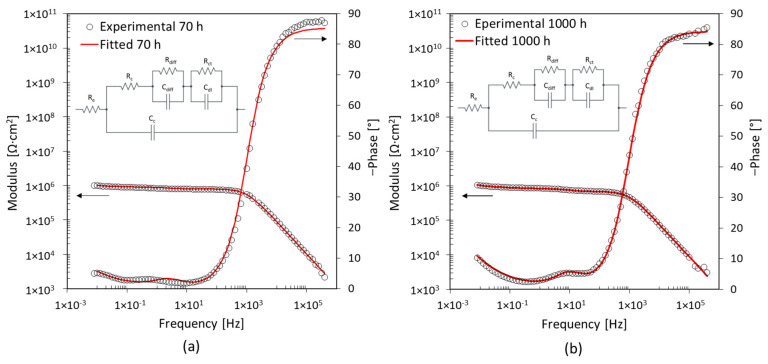
EIS spectra and EEC after (**a**) 70 h and (**b**) 1000 h exposure for specimen C2 with 3 wt% of nanoparticles.

The last type of behaviour contains those samples that had on the surface evident corrosion products and numerous areas with detachment of the coating from the substrate, shown in Figure 4e–g. The most representative specimen is shown below, in Figure 14. In this case, no significant changes were found during the entire exposure time, as shown in Table 5. The EEC used remained unchanged. The low value of the impedance modulus in the high-frequency zone (≈10^3^ Hz) indicates an immediate penetration of water under the coating through preferential paths, as reported by Hinderliter et al. [35]. This is confirmed by the low value of R_c_, as reported in Table 5. The middle-frequency time constant (R_diff_ and C_diff_) is related to the formation of deposits of corrosion products on the base metal [37]. The low-frequency time constant (R_ct_ and C_dl_) is attributed to the interfacial charge transfer reaction.

The addition of nanoparticles did not improve the barrier properties of the coating, which seems to show even worse behaviour than in the case of the coating with 0 wt% of nano clays.

The use of EECs makes it possible to understand, through their variation, how the coating behaves throughout a test period. All the elements that can be used in these circuits can be linked to physical phenomena that occur in the coating or at the interface with the substrate. Among these, there is one element that, in addition to C_c_, can be used to assess the effect of nanoparticles on the barrier properties of the coating. This parameter is the coating resistance, R_c_, which provides an understanding of the protection exercised by the coating. R_c_, from a physical point of view, can be associated with the coating’s efficiency in hindering the penetration of water inside it [34]. The value of R_c_ can be used to estimate the electrical resistivity, ρ, of each coating throughout the exposure period and compare it with the electrical resistivity of epoxy resin, ρ_e_ = 2 × 10^11^ Ω∙cm^2^ [35]. This parameter represents the ideal resistance of the coating, considered without defects.

**Figure 15:**
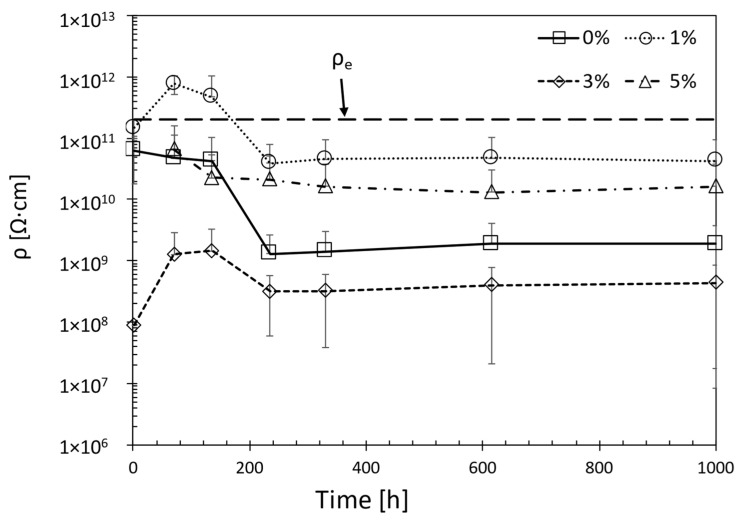
Electrical resistivity, ρ, variation over time compared with the value of ρ_e_.

Figure 15 shows the time course of the electrical resistivity of each specimen analysed previously compared with the theoretical value of the electrical resistivity of epoxy resin. The value of electrical resistivity, ρ, and standard deviation of electrical resistivity, σ_ρ_, of the specimens are available in the Supplementary Materials.

The value of ρ_e_ is the resistivity of a coating in perfect condition, free of defects, and thus able to protect the substrate. Therefore, when the resistivity falls below this value, corrosion initiation can be assumed. The EEC analysis confirms this assumption.

The specimen with 1 wt% nanofiller shows the second-time constant, associated with the corrosion process, after 135 h of exposure, as shown in Figure 9b. The development of the parameter ρ confirms that, after the corrosion initiation, the resistance associated with the pores of the coating decreases. Despite this, in the case of specimens with 1 wt% of nano clay, the value of ρ remains high, settling at 4.50 × 10^10^ Ω∙cm. The lack of evident corrosion products, in one case (Figure 4c), and the presence of a single slight corrosive attack in the second case (Figure 4d), testify that nanoparticles within the epoxy resin allowed the creation of more tortuous paths for water penetration. For the coating without nanoparticles, the ρ decayed by about two orders of magnitude, as shown in Figure 15. Moreover, already after 2 h of exposure, this parameter is below the ρ_e_ threshold value. This coating is not able to hinder the penetration of water into it. This is also evidenced by the analysis of the specimen surface (Figure 4a,b) and EIS spectra (Figure 12 and Figure 13).

**Figure 16:**
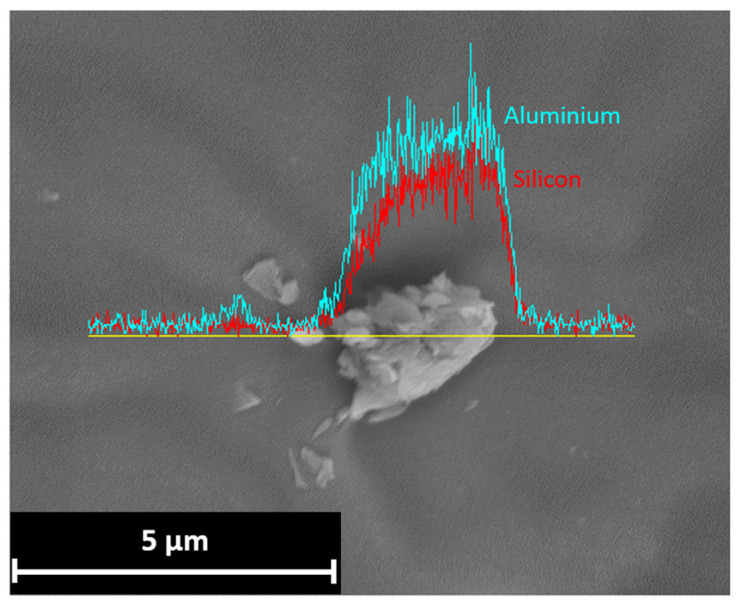
EDS analysis of a nano clay cluster in the coating with 3 wt% addition of Cloisite 30B^®^.

For higher concentrations than 1 wt%, the improved effect is related to the dispersion of the nanoparticles. In the case of the specimen with 3 wt% nanofiller, the ρ value was even lower than that for the epoxy resin coating alone, as shown in Figure 15. The strong tendency to form clusters generated an effect contrary to the desired one. From the very first moments of the test, water penetrated the coating through the formation of preferential paths. This led to increased water absorption and the formation of obvious corrosion products. The cluster formation is mainly linked to two factors: an incorrect dispersion procedure of the nanofillers and an increasing concentration of dispersed particles within the coating [42,43,44]. The EDS analysis performed on the C2 coating at the end of the test shows the presence of these clusters within the coating, shown in Figure 16. Their micrometric size caused an early degradation of the barrier properties, promoting the penetration of water and subsequent corrosion of the substrate.

For even more increased additions than 3 wt%, there is a two-factor behaviour of the coating. The tendency to form clusters causes a worsening of the barrier properties, as evidenced by the trend in ρ, shown in Figure 15. At the same time, the increase in concentration allows the dispersion of more nanoparticles. This factor favours the creation of more tortuous paths for water penetration, resulting in better barrier properties. The higher ρ value compared to the 3 wt% nanofiller specimen, and the much lower surface corrosion, confirms this improvement.

## 4. Conclusions

In conclusion, the technique of electrochemical impedance spectroscopy combined with the processing of the results using equivalent electrical circuits allows the evolution of the protective capacity of coatings to be understood over time.

The addition of nanoparticles improves the barrier properties of the epoxy resin coating alone. This improvement of barrier properties can already be observed for low additions of 1 wt% but is linked to the proper dispersion of the nanoparticles.

For additions higher than 1 wt%, the barrier properties are affected in two ways. The presence of macro-clusters increases with increasing nano clay concentration and reduces its protective capacity. At the same time, increasing the concentration of nanoparticles allows for more dispersed nanofillers, raising the barrier properties of the coating.

## Figures and Tables

**Table 1:** Specimen designations and wet and dry thickness of coatings.

	Test Conditions							
Nano clay addition [wt%]	0%		1%		3%		5%	
Specimen	A1	A2	B1	B2	C1	C2	D1	D2
Wet thickness [µm]	75–100	75–100	75–100	75–100	50–75	75–100	75–100	50–75
Dry thickness [µm]	55 ± 3	52 ± 3	55 ± 8	56 ± 6	39 ± 7	52 ± 5	55 ± 9	44 ± 3

**Table 2:** Fitting results obtained for specimen B1.

Time [h]	R_s_ [Ω∙cm^2^]	R_c_ [Ω∙cm^2^]	C_c_ [F/cm^2^]	nC_c_	R_ct_ [Ω∙cm^2^]	C_dl_ [F/cm^2^]	nC_dl_	Z_w_ [F/cm^2^]
2	20	1 × 10^9^	2 × 10^−10^	0.96	-	-	-	-
70	20	5 × 10^9^	1 × 10^−10^	0.95	-	-	-	-
135	20	4 × 10^8^	2 × 10^−10^	0.97	1 × 10^8^	9 × 10^−9^	0.80	1 × 10^−8^
235	20	4 × 10^8^	2 × 10^−10^	0.96	1 × 10^8^	7 × 10^−9^	0.62	-
330	20	4 × 10^8^	2 × 10^−10^	0.96	1 × 10^9^	4 × 10^−9^	0.58	-
615	20	5 × 10^8^	3 × 10^−10^	0.96	9 × 10^8^	4 × 10^−9^	0.68	-
1000	20	4 × 10^8^	2 × 10^−10^	0.96	1 × 10^9^	4 × 10^−9^	0.60	-

**Table 3:** Fitting results obtained for specimen D2.

Time [h]	R_s_ [Ω∙cm^2^]	R_c_ [Ω∙cm^2^]	C_c_ [F/cm^2^]	nC_c_	R_ct_ [Ω∙cm^2^]	C_dl_ [F/cm^2^]	nC_dl_	R_diff_ [Ω∙cm^2^]	C_diff_ [F/cm^2^]	nC_diff_
70	20	6 × 10^8^	2 × 10^−10^	0.95	8 × 10^8^	5 × 10^−9^	0.60	-	-	-
135	20	2 × 10^8^	3 × 10^−10^	0.95	3 × 10^8^	5 × 10^−8^	0.8	3 × 10^8^	5 × 10^−9^	0.77
235	20	2 × 10^8^	2 × 10^−10^	0.94	3 × 10^8^	4 × 10^−8^	0.65	6 × 10^8^	5 × 10^−9^	0.78
330	20	1 × 10^8^	3 × 10^−10^	0.95	2 × 10^8^	4 × 10^−8^	0.65	2 × 10^8^	4 × 10^−9^	0.73
615	20	1 × 10^8^	3 × 10^−10^	0.95	2 × 10^8^	4 × 10^−8^	0.65	2 × 10^8^	4 × 10^−9^	0.74
1000	20	1 × 10^8^	3 × 10^−10^	0.96	1 × 10^8^	7 × 10^−8^	0.7	2 × 10^8^	5 × 10^−9^	0.74

**Table 4:** Fitting results obtained for specimen A2.

Time [h]	R_s_ [Ω∙cm^2^]	R_c_ [Ω∙cm^2^]	C_c_ [F/cm^2^]	nC_c_	R_ct_ [Ω∙cm^2^]	C_dl_ [F/cm^2^]	nC_dl_	R_diff_ [Ω∙cm^2^]	C_diff_ [F/cm^2^]	nC_diff_
2	20	2 × 10^8^	4 × 10^−10^	0.95	7 × 10^9^	5 × 10^−9^	0.58	-	-	-
70	20	4 × 10^8^	4 × 10^−10^	0.94	4 × 10^9^	5 × 10^−9^	0.62	-	-	-
135	20	4 × 10^8^	4 × 10^−10^	0.96	2 × 10^9^	3 × 10^−9^	0.58	-	-	-
235	20	2 × 10^6^	4 × 10^−10^	0.93	9 × 10^6^	7 × 10^−8^	0.61	-	-	-
330	20	1 × 10^6^	4 × 10^−10^	0.95	1 × 10^7^	7 × 10^−8^	0.60	-	-	-
615	20	1 × 10^6^	4 × 10^−10^	0.95	1 × 10^7^	7 × 10^−8^	0.56	-	-	-
1000	20	2 × 10^6^	4 × 10^−10^	0.95	1 × 10^7^	2 × 10^−7^	0.58	5 × 10^6^	1 × 10^−8^	0.76

**Table 5:** Fitting results obtained for specimen C2.

Time [h]	R_s_ [Ω∙cm^2^]	R_c_ [Ω∙cm^2^]	C_c_ [F/cm^2^]	nC_c_	R_ct_ [Ω∙cm^2^]	C_dl_ [F/cm^2^]	nC_dl_	R_diff_ [Ω∙cm^2^]	C_diff_ [F/cm^2^]	nC_diff_
70	20	9 × 10^5^	3 × 10^−10^	0.95	3 × 10^5^	3 × 10^−5^	0.80	9 × 10^4^	2 × 10^−6^	0.97
135	20	7 × 10^5^	3 × 10^−10^	0.95	3 × 10^5^	4 × 10^−5^	0.70	7 × 10^4^	7 × 10^−7^	0.96
235	20	7 × 10^5^	3 × 10^−10^	0.95	6 × 10^5^	3 × 10^−5^	0.67	8 × 10^4^	6 × 10^−7^	0.96
330	20	6 × 10^5^	4 × 10^−10^	0.94	6 × 10^5^	2 × 10^−5^	0.62	1 × 10^5^	3 × 10^−7^	0.97
615	20	7 × 10^5^	5 × 10^−10^	0.93	7 × 10^5^	2 × 10^−5^	0.60	9 × 10^4^	2 × 10^−7^	0.97
1000	20	7 × 10^5^	5 × 10^−10^	0.94	8 × 10^5^	1 × 10^−5^	0.60	1 × 10^5^	1 × 10^−7^	0.98

## Data Availability

The original contributions presented in the study are included in the article/Supplementary Materials, further inquiries can be directed to the corresponding author/s.

## Supplementary Materials

The following supporting information can be downloaded at: https://www.mdpi.com/article/10.3390/ma17123028/s1, Figure S1: Nyquist plot after (a) 2 h and (b) 235 h and 1000 h exposure for specimen A2 with 0 wt% of nanoparticles; Figure S2: Nyquist plot after (a) 2 h and 135 and (b) 235 h and 1000 h exposure for specimen B1 with 1 wt% of nanoparticle; Figure S3: Nyquist plot after 70 h and 1000 h exposure for specimen C2 with 3 wt% of nanoparticles; Figure S4: Nyquist plot after 70 h, 135 h and 1000 h exposure for specimen D2 with 5 wt% of nanoparticles; Table S1: Electrical resistivity, ρ, and standard deviation of electrical resistivity, σ_ρ_, of the specimens.
