# Supplementary material for: Electrochemical Impedance Spectroscopy Analysis of Organic Epoxy Coatings Reinforced with Nano Clay
Source: Materials (Basel). 2024 Jun 20;17(12):3028. doi: 10.3390/ma17123028 (PMC11205866; doi:10.3390/ma17123028)
Supplement: Supplementary file 1 [file materials-17-03028-s001.zip › materials-3025891-supplementary.pdf]

materials

MDPI

Article

Electrochemical Impedance Spectroscopy Analysis of Organic Epoxy Coatings Reinforced with Nano Clay

Davide Asperti [1,*], Marina Cabrini [1,*], Sergio Lorenzi [1], Giuseppe Rosace [1], Abdollah Omrani [2] and Tommaso Pastore [1]

[1] Department of Engineering and Applied Sciences, School of Engineering, University of Bergamo, 24044 Dalmine, Italy; sergio.lorenzi@unibg.it (S.L.); giuseppe.rosace@unibg.it (G.R.); tommaso.pastore@unibg.it (T.P.)
[2] Faculty of Chemistry Iran, University of Mazandaran, Babolsar 4741613534, Iran; omrani@umz.ac.ir
* Correspondence: davide.asperti@unibg.it (D.A.); marina.cabrini@unibg.it (M.C.)

Citation: Asperti, D.; Cabrini, M.; Lorenzi, S.; Rosace, G.; Omrani, A.; Pastore, T. Electrochemical Impedance Spectroscopy Analysis of Organic Epoxy Coatings Reinforced with Nano Clay. *Materials* **2024**, *17*, 3028. https://doi.org/10.3390/ma17123028

Academic Editor: Dimitrios Papoulis

Received: 8 May 2024
Revised: 15 June 2024
Accepted: 17 June 2024
Published: 20 June 2024

Copyright: © 2024 by the authors. Licensee MDPI, Basel, Switzerland. This article is an open access article distributed under the terms and conditions of the Creative Commons Attribution (CC BY) license (https://creativecommons.org/licenses/by/4.0/).

1. Supplementary results

Table S1. Electrical resistivity, ϱ, and standard deviation of electrical resistivity, σ_{ϱ}, of the specimens.

	0%		1%		3%		5%	
**Time [h]**	**ϱ [Ω·cm]**	**σ_{ϱ} [Ω·cm]**	**ϱ [Ω·cm]**	**σ_{ϱ} [Ω·cm]**	**ϱ [Ω·cm]**	**σ_{ϱ} [Ω·cm]**	**ϱ [Ω·cm]**	**σ_{ϱ} [Ω·cm]**
2	6.3×10^{10}	4.6×10^{10}	1.5×10^{11}	5.2×10^{10}	8.9×10^{7}	-	-	-
70	4.8×10^{10}	6.6×10^{10}	7.8×10^{11}	2.5×10^{11}	1.3×10^{9}	1.6×10^{9}	6.6×10^{10}	9.3×10^{10}
135	4.3×10^{10}	5.8×10^{10}	4.6×10^{11}	5.6×10^{11}	1.4×10^{9}	1.9×10^{9}	2.3×10^{10}	3.2×10^{10}
235	1.3×10^{9}	1.4×10^{9}	3.9×10^{10}	4.2×10^{10}	3.2×10^{8}	2.6×10^{8}	2.1×10^{10}	2.9×10^{10}
330	1.4×10^{9}	1.6×10^{9}	4.6×10^{10}	4.8×10^{10}	3.3×10^{8}	2.9×10^{8}	1.6×10^{10}	2.1×10^{10}
615	1.9×10^{9}	2.3×10^{9}	4.7×10^{10}	5.4×10^{10}	4.0×10^{8}	3.8×10^{8}	1.3×10^{10}	1.7×10^{10}
1000	1.9×10^{9}	1.9×10^{9}	4.3×10^{10}	5.2×10^{10}	4.4×10^{8}	4.3×10^{8}	1.6×10^{10}	2.2×10^{10}

Nyquist plot

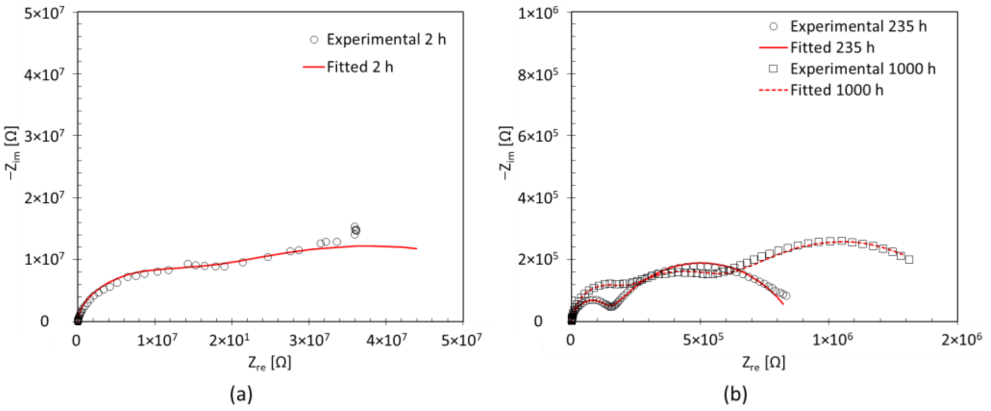

Figure S1. Nyquist plot after (a) 2 h and (b) 235 h and 1000 h exposure for specimen A2 with 0 wt% of nanoparticles.

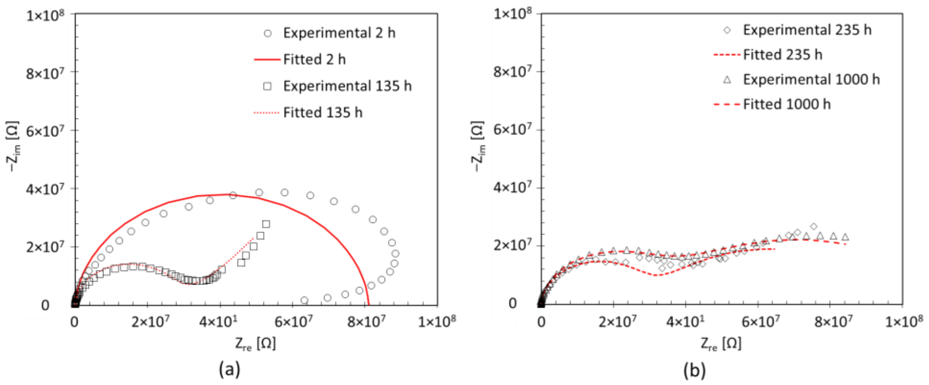

Figure S2. Nyquist plot after (a) 2 h and 135 and (b) 235 h and 1000 h exposure for specimen B1 with 1 wt% of nanoparticle.

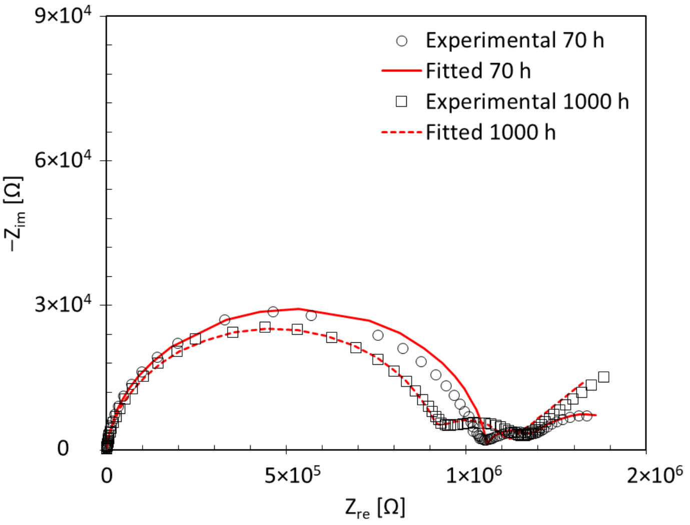

Figure S3. Nyquist plot after 70 h and 1000 h exposure for specimen C2 with 3 wt% of nanoparticles.

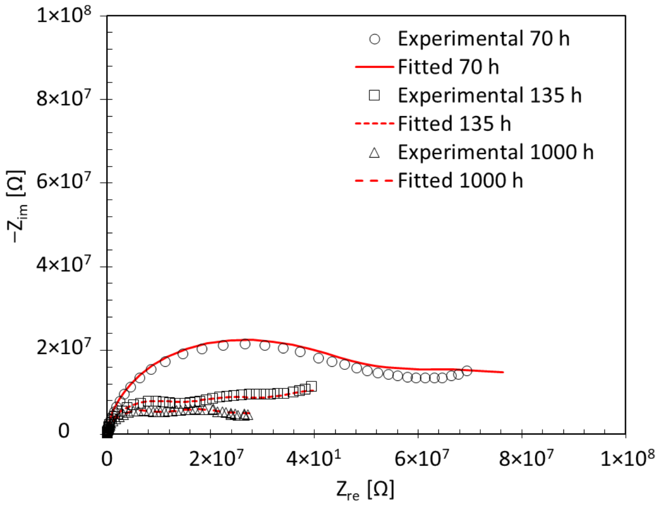

Figure S4. Nyquist plot after 70 h, 135 h and 1000 h exposure for specimen D2 with 5 wt% of nanoparticles.
